# *Mycobacterium orygis* and its unseen impact: re-evaluating zoonotic tuberculosis in animal and human populations

**DOI:** 10.3389/fpubh.2025.1505967

**Published:** 2025-03-21

**Authors:** Indu Rani, Rakesh Kumar, Harisankar Singha, Thachamvalley Riyesh, Rajesh Kumar Vaid, Tarun Kumar Bhattacharya, Karuppusamy Shanmugasundaram

**Affiliations:** ^1^ICAR-National Research Centre on Equines, Hisar, Haryana, India; ^2^Department of Microbiology, College of Basic Sciences and Humanities, Chaudhary Charan Singh Haryana Agricultural University, Hisar, Haryana, India

**Keywords:** zoonotic tuberculosis, *Mycobacterium orygis*, neglected pathogen, One Health, surveillance, pathogenicity

## Abstract

Globally, the incidence and burden of zoonotic tuberculosis (zTB) in humans are underestimated. Earlier, it was considered that *Mycobacterium bovis* (*M. bovis*) was the sole etiology of zTB; however, novel zoonotic species of mycobacteria, namely, *Mycobacterium orygis* (*M. orygis*), is also implicated and often neglected pathogen, which necessitates more attention. *M. orygis* has been recently included under the members of the *Mycobacterium tuberculosis* complex (MTBC), and it shares genetic similarities with other members of this complex. *M. orygis* can cause tuberculosis (TB) in animals and humans. This bacterium is harbored by a wide range of host species; however, the exact host spectrum is not well understood. In recent years, *M. orygis* has received considerable interest due to its frequent isolation in zTB infections that often originated from tuberculosis-endemic countries than non-endemic countries. Therefore, the zoonotic potential of this bacterium highlights the importance of “One Health” approaches in understanding its possible routes of transmission, reservoir, ecology, and pathogenicity. Moreover, the occurrence of *M. orygis* in tuberculosis-endemic countries with limited resources poses further challenges in disease surveillance and identification, emphasizing the significance of collaborative measures across multiple sectors to monitor and control its spread. This review focuses on the current knowledge of *M. orygis* and underscores the importance of this neglected pathogen, which has potential impacts on both human and animal health.

## Introduction

Tuberculosis (TB) is a pandemic infectious disease and poses an important threat to global public health (1). Indeed, millions of people worldwide lose their lives to TB every year (2). *Mycobacterium tuberculosis* complex (MTBC) is responsible for TB in both humans and animals (3), and MTBC consists of well-defined members of *Mycobacterium* species such as *M. tuberculosis*, *Mycobacterium bovis*, *Mycobacterium caprae*, *Mycobacterium pinnipedii*, *Mycobacterium africanum*, *Mycobacterium mungi*, *Mycobacterium canettii*, and *Mycobacterium microti* (4). All species in this group have identical nucleotides (99.9%) at their genome level and have homogeneity in their 16S rRNA sequences (5). However, they differ from each other by their phenotypic characteristics, host spectrum, pathogenicity, and disease epidemiology (6).

Among the MTBC, TB due to the oryx bacillus has been recorded in oryx and other animals. This name conveys the fact that this pathogen was primarily isolated from antelopes, a member of the *Bovidae* family (7, 8). Oryx bacillus was considered a phenotypically divergent subtype of *M. bovis* due to the sharing of a unique spoligotype pattern with this species (9). For instance, spoligotyping patterns and *IS6110* copy numbers of oryx bacilli, namely, Kremer 24 and Kremer 69 isolates, were matched with *M. bovis*. On the other hand, these two isolates had five specific deletions (RDoryx_wag22, RDoryx_1, RD5oryx, RD12oryx, and RDoryx_4), and these deletions were absent in other members of MTBC. Therefore, oryx bacilli were considered to be genetically different from *M. bovis* strains and deserve an exclusive phylogenetic position within the MTBC (6). In 2012, 22 oryx bacilli isolated from different hosts were analyzed by restriction fragment length polymorphism (RFLP) targeting *IS6110* gene sequences, region of difference (RD), and single-nucleotide polymorphism (SNP) and renamed this zoonotic pathogen as *M. orygis*, which is now considered as a new subspecies under MTBC with a distinct lineage (4).

Though there are currently no reliable estimates based on thorough surveys to evaluate the impact that zTB poses to the health of humans and animals (10), the impact of zTB is wide-ranging and multifaceted, affecting both humans and animals. TB in animals represents a threat to animal health, leading to chronic disease, economic loss due to decreased productivity, trade restrictions, challenges in disease surveillance, and costs involved in the implementation of control programs (11). It would be very expensive and difficult to eradicate zTB from wild animals, and these animals may act as a source of infection to the entire ecosystem (12). Deforestation and rapid urbanization increase contact between wild animals, livestock, and humans, and as a result of repercussions, many diseases, including zTB, may increase in the future. Nonetheless, it is evident that the existence of zTB in livestock and wildlife populations affects human health and food safety (10). *M. orygis* can cause TB in animals but also affects humans (13). The true burden of *M. orygis* and its zoonotic potential remains unknown worldwide. However, based on scientific literature, it seems that the number of *M. orygis* cases has increased in recent years. Advances in genome sequencing technologies and bioinformatics tools have made it possible to identify members of the MTBC up to the subspecies level, which was not feasible in the past by traditional laboratory techniques (14). This could be a reason for the increased reporting of *M. orygis* cases nowadays. Therefore, increased awareness among physicians, veterinarians, laboratories, and public health personnel under ‘One-Health’ approach is imperative to understand the epidemiology, transmission cycle, host spectrum, reservoirs, and zoonotic potential of *M. orygis.* This review aims to highlight the challenges and threats of this neglected *M. orygis* for humans, animals, and the environment.

## Characteristics of *M. orygis* and genetic variations between *M. orygis* and other members of the MTBC

*Mycobacterium orygis* is a very slow-growing, acid-fast bacterium with an incubation period of 5–8 months. Its colony morphology is smooth to greasy, moist, non-chromogenic, and domed-shaped on solid media (15, 16). Information on the specific phenotypic and biochemical characteristics of *M. orygis* is scarce and requires further study. The whole-genome sequencing of *M. orygis* (strain NIRTAH144) reveals that it has a ~4.29 Mb genome with 65.59% GC content (17). Complete genome sequencing of another *M. orygis* (strain 51,145) yielded 4,352,172 bp and a 65.6% GC content (18). Recently, human (*n* = 322)- and animal (*n* = 529)-adapted MTBC were analyzed by maximum likelihood topology and thus placed *M. orygis* under animal-adopted MTBC clade A3 (19). This phylogenetic study suggested that *M. orygis* shares an ancestor that is common to *M. bovis* and *M. caprae* (19). Another study proposed that it should be positioned below the *M. africanum* but above the *M. bovis* and *M. caprae* in phylogeny (9). However, its exact phylogenetic position remains unsettled. Since *M. orygis* and the other members of MTBC are closely linked at the genomic level, identifying and differentiating them from one another using traditional approaches becomes a challenging task, which led to a paucity of data regarding the clinical characteristics of *M. orygis*.

Genotyping studies reveal that there are genomic insertions and deletions in the members of MTBC; these large sequence polymorphisms are known as RD and are used for the differentiation of the MTBC (20). Comparative genomic studies showed that there is a genomic insertion of 2–12.7 Kb in the *M. tuberculosis* H37Rv strain, and this region is absent in other species of MTBC. Thus, the deletion of this RD creates diversity among other members of MTBC (21). Recently, RD motif analysis by PCR-based techniques such as single tube multiplex qPCR has been explored to discriminate the members of MTBC (9, 22), and the findings are shown in Table 1. According to the RD analysis, *M. orygis* has RD1 and RD4 and lacks RD7, RD8, RD9, RD10, and RD12 (4, 17). Single-nucleotide polymorphisms in the *gyrB*, *pncA, oxyR, leuS, mmpS6, PPE55, Rv0444c,* and *hsp* genes of MTBC are also useful for discriminating between MTBC species (23–25). *M. orygis* shows specific nucleotide transitions such as T to G at the 38th codon of *Rv2042C*, G to C at the 698th codon of *Rv0444c*, C to G at the 551st codon of *mmpL6*, the C to T position in *PPE55*, and other SNPs (4, 9, 24, 26). Of these, a novel GGC mutation in the *Rv2042*^38^ gene is a specific SNP marker for *M. orygis* (Table 2). However, other researchers claim that the SNP at the 1,113 (G to A) position of the *gyrB* gene is a more useful marker to discriminate *M. orygis* from other MTBC species (27).

**Table 1:** Differentiation of *Mycobacterium tuberculosis* complex (MTBC) using region of differences-PCR analysis (9, 22).

*Mycobacterium tuberculosis* complex (MTBC)	Region of differences			
	RD1	RD4	RD9	RD12
*M. tuberculosis* (H37Rv)	+	+	+	+
*M. bovis*	+	−	−	−
*M. bovis BCG*	−	−	−	−
*M. caprae*	+	+	−	−
*M. orygis*	+	+	−	−
*M. africanum*	+	+	−	+
*M. microti*	−	+	−	+
*M. canettii*	+	+	+	−

**Table 2:** Single-nucleotide polymorphism (SNP) analysis for differentiation *Mycobacterium tuberculosis* complex (MTBC) (4, 9, 24, 26).

MTBC	*hsp65*	*oxyR*	*pncA*	*mmpL6*		*mmpS6*	*leuS*		*TbD1*	*Rv2042c*		*Rv0444c*			*gyrB*								*PPE55*	
	631	285	169	486	551	334	1,064	1,251	171	113	38	320	551	698	277	675	756	870	1,113	1,410	1,450	1,478	2,162	2,163
*M. tuberculosis*	C	G	C	T	C	C	A	G	C	T	T	C	C	G	A	C	G	A	G	C	G	T	T	C
*M. bovis*	C	A	G	T	G	C	A	G	C	T	T	T	T	G	A	C	A	G	G	T	T	T	T	C
*M. bovis BCG*	C	A	G	T	G	C	A	G	C	T	T	T	T	G	A	C	A	G	G	T	T	T	T	C
*M. africanum*	C	G	C	T	C	C	A	G	C	T	T	C	C	G	A	C	G	G	G	C	T	T	T	C
*M. orygis*	C	G	C	C	G	G	T	T	G	G	G	C	C	C	G	C	G	G	A	C	T	C	G	T

## Host spectrum

In 1976, *M. orygis* was first reported in two East African oryx and later in 1987 from an oryx kept in a zoo in the Netherlands. Thereafter, *M. orygis* has been identified as a potential pathogen for members of the *Bovidae* family (28). Nowadays, it has been reported in a variety of animals, such as African buffalo, domestic cattle, gazelles, waterbucks, deer, rhinoceros, and blue bulls (7, 13, 28). Until now, a vast number of *M. orygis* cases have been reported from human patients suffering from TB (19), and it has been reported from rhesus monkeys (26, 29). It is necessary to differentiate the maintenance host from the spillover host to identify *M. orygis* host tropism. Some of the studies suggest that cattle are the maintenance host for *M. orygis*, and other animals are considered to be spillover hosts, which are infected with *M. orygis* due to contact with infected cattle. Some researchers also speculated that *M. orygis* and *M. bovis* were adapted to *Bos indicus* and *Bos taurus*, respectively (19). Nevertheless, further study is required to identify the precise host range of *M. orygis* (28).

## Disease transmission

**Figure 1:**
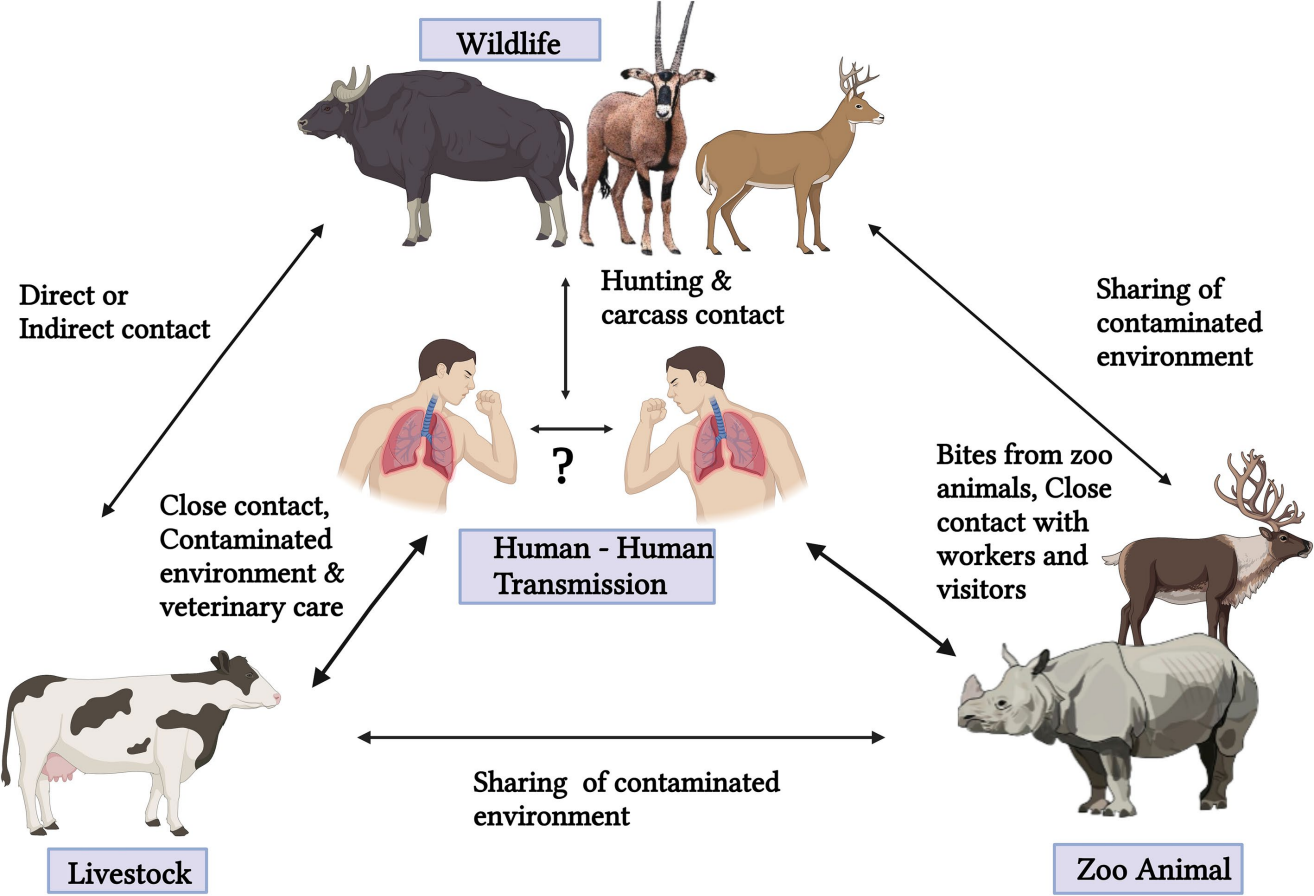
Transmission cycle of *Mycobacterium orygis.*

The exact route of transmission of *M. orygis* is not ruled out yet. Recent studies suggest that aerosol transmission is the most common route in disease spread (26). Other possible routes are through the ingestion of *M. orygis*-contaminated water and food, as well as vertical and cutaneous transmission (Figure 1). Close contact with infected animals and consumption of raw milk and undercooked meat of infected animals could also act as a source of disease transmission to humans (15, 30–32). There is a possibility that *M. orygis* may spread from human to human, and it needs more epidemiological studies to understand the transmission route of this pathogen (29).

## Clinical signs and gross pathology

Specific clinical symptoms and incubation periods of *M. orygis* are poorly understood and not well documented yet. However, in general, infected humans typically show clinical symptoms such as fever, dry cough, body pain, loss of appetite, dysuria, loss of body weight, nausea, and lymph node enlargement (33). Infected animals may exhibit non-specific clinical signs such as nasal discharge, dullness and depression, sneezing, anorexia, weakness, cough, diarrhea, gradual weight loss, pneumonia, enlarged lymph nodes, and dyspnea (13, 26, 31). Sometimes, in the environment, performing a postmortem examination is not possible considering the risk of contamination of the environment and spreading of organisms to other susceptible hosts (34). Thus, this limits the availability of comprehensive descriptive tuberculous lesions caused by *M. orygis* (15). Based on the limited information on the gross pathology, in most of the cases, granulomatous lesions were observed in the lungs, lymph nodes, liver, and gastrointestinal tract of animals and are responsible for both pulmonary and extra-pulmonary TB (13, 26, 30, 35, 36). Pathological findings in a wild African buffalo showed a focal well-encapsulated pulmonary granuloma in the lower half of the dorso-caudal portion of the right cranial lung lobe. Below the capsule, an area of apparent calcification was also observed and a cut section of the granuloma had a central caseous necrosis with a partial liquefaction. In a deer, both extra-pulmonary and pulmonary tuberculous nodules of varying sizes (1–2 cm) were noticed; these nodules were encapsulated and liquefied in nature (15). In another study, calcified, pale-yellow lesions were recorded in the lungs and lymph nodes of blackbucks and spotted deer (37). Neural granulomatosis was also reported in a recent study (38). Multifocal, single-to-coalescing calcified caseous nodules were seen in the lungs and lymph nodes of the buffalo, cattle, and deer and in the liver of cattle infected with *M. orygis* (39).

## Treatment

The treatment regimen for TB is outside the scope of this review. However, various treatment options are available, depending on the drug sensitivity and resistance of the TB infection, and these guidelines are regularly updated by the WHO. In general, a combination of isoniazid, rifampin, pyrazinamide, and ethambutol for drug-sensitive TB is considered the first-line treatment for TB (40), and the duration varies between 4 and 6 months (41). For drug-resistant TB cases, repurposed drugs such as moxifloxacin, levofloxacin, beta-lactams, linezolid, and clofazimine, as well as newer drugs such as delamanid, bedaquiline, and pretomanid, are commonly used (40). The treatment duration for drug-resistant TB ranges from 6 to 18 months (42). According to Centre for Disease Control (CDC) guidelines, latent TB can be treated with (i) isoniazid and rifapentine, once weekly for 3 months; (ii) isoniazid and rifampin, daily for 3 months; (iii) rifampin, daily for 4 months; or (iv) isoniazid, daily for 6–9 months or twice weekly for 52–76 weeks (43). Treatment for TB caused by *M. orygis* in humans is similar to that provided for TB caused by *M. tuberculosis*, and it generally lasts from 9 months to 2 years (33). For more detailed information on TB treatment, readers can refer to the WHO and CDC guidelines. In animals, TB infection can be treated with the same drugs used in humans; however, no systematic studies have been conducted to evaluate the efficacy of these drugs in eliminating TB agents (44).

## Origin and geographical distribution of *M. orygis* infection in animals

**Figure 2:**
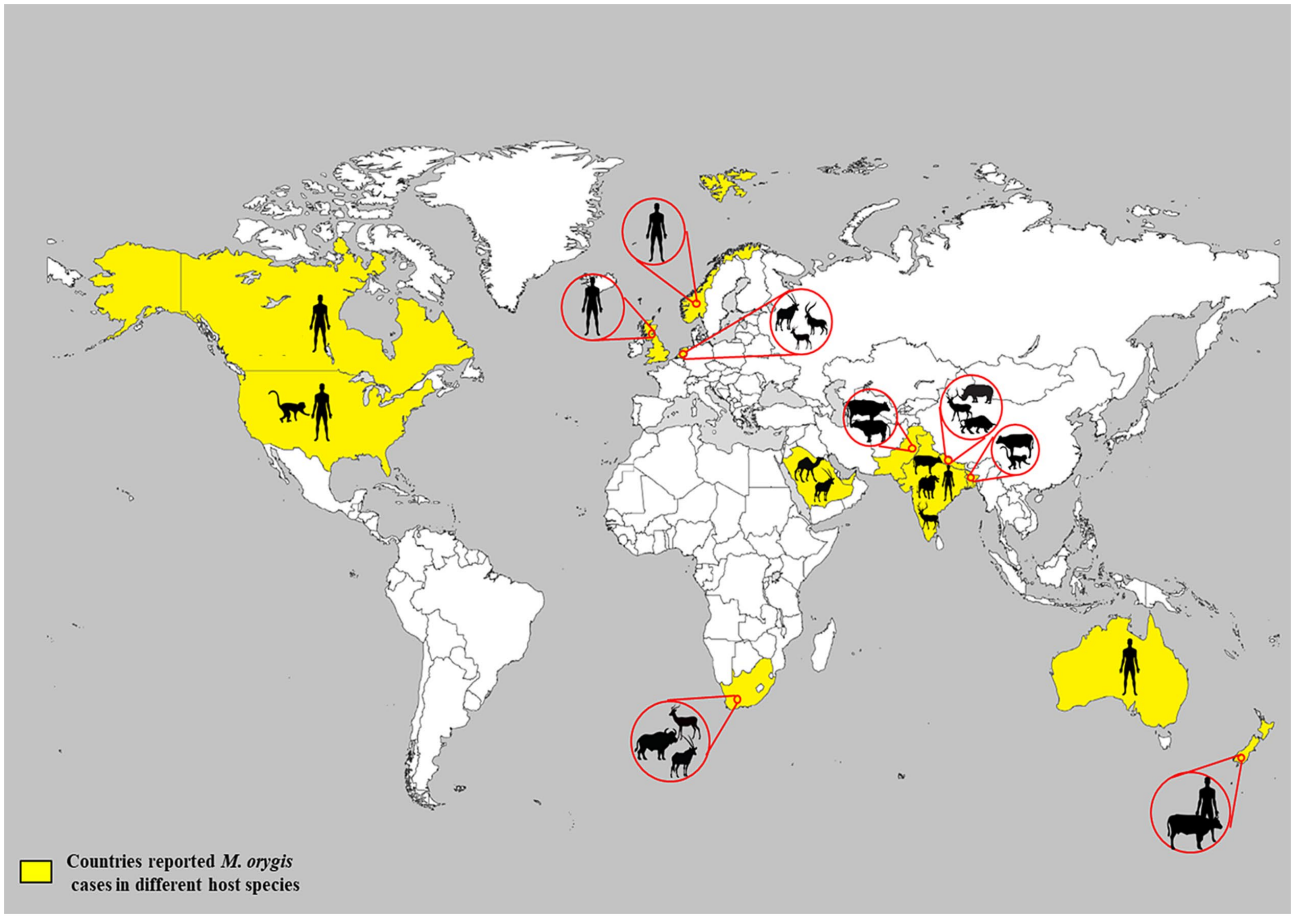
Global reporting of *M. orygis* infection in animals and humans.

Due to a lack of systematic surveillance, the global prevalence of TB caused by *M. orygis* is unknown. Various molecular and epidemiological studies on MTBC strains indicated that *M. orygis* diverges phylogenetically from other MTBC members (4, 45). It has been postulated that with the migration of humans and the subsequent global spread of MTBC, members could have resulted in the introduction of distinct MTBC subspecies into various geographic regions at different rates (26). Hence, *M. orygis* may have dispersed to South Asia before the arrival of *M. bovis* and evidence in past studies suggested that *M. orygis* might be endemic in South Asian countries (4, 26, 45). Later, due to globalization, urbanization, and international trade of animals and their products, the disease has spread to various regions of the world. Based on the limited information (Table 3), infections have been reported in both wild and domestic animals in South Asia, Africa, and the Arabian Peninsula (46) (Figure 2). Between 1987 and 1988, *M. bovis* strains isolated from waterbuck, oryx, and antelopes in a single zoo in the Netherlands were characterized by *IS6110*-associated RFLP. These isolates had a highly unusual copy number of *IS6110* bands (*n* = 20), and later on, some of these isolates were re-identified as *M. orygis* (4, 7). Saudi Arabia had an outbreak in Arabian oryx in 1994, and possibly, this outbreak was caused by *M. orygis* (27, 47). In the United Arab Emirates, mycobacteria were isolated from dromedary camels suffering from TB and gene deletion analysis showed that these isolates lacked RD7, RD8, and RD9. Furthermore, SNP studies of the *mmpL6* gene of the isolates revealed the presence of a G residue in the 551 codon position, and these properties were matched with members of the antelope clade of *M. bovis* (35, 48). The authors suspected that the ingestion of bacilli excreted from the gazelle could be a possible route for transmission to camels. Recently, in Southern Africa, a free-ranging African buffalo (*Syncerus caffer*) was found to be positive for TB by three consecutive comparative intradermal tuberculin tests. Subsequently, *M. orygis* was isolated from a lung sample and confirmed by comprehensive molecular assays (27). In 2024, the United States reported the presence of *M. orygis* in 26 *Macaca fascicularis* (*Cynomolgus macaques*) that were imported from Southeast Asia for scientific studies (49).

**Table 3:** Global status of *M. orygis* differentiated from other MTBC isolates.

Country	Host	Total Number of MTBC isolates	*M. orygis* isolates	References
Asia				
India	Spotted deer and blackbucks	3	3	37
India	Bulls, bull calves, and bullocks	16	13	51
India	Spotted deer and Indian bison	32	3	50
India	Humans	940	7	45
Nepal	Spotted deer and blue bull	2	2	15
Pakistan	Cattle and buffalo	20	10	34
Bangladesh	Cattle and rhesus monkey	20	20	26
Bangladesh	Cattle	4	3	4, 36
United Arab Emirates	Camels	3	3	35, 48
North America				
United States (New York)	Human	6,323	9	30
United States	Monkey	26	26	49
Canada	Human	3,599	21	25
Europe				
United Kingdom	Human	3,128	24	14
Netherlands	Waterbuck, antelope, and oryx	13	6	4, 7
Norway	Human	5	5	56
Australia and Oceania				
Australia	Human	1763	8	55
New Zealand	Cattle	1	1	29
New Zealand	Human	1	1	29
Africa				
South Africa	African buffalo	1	1	27

In South Asia, most of the cases were reported from India, Nepal, Pakistan, and Bangladesh (Table 3), and it has been proposed that *M. orygis* is endemic in these countries (29, 34) (Figure 2). A study conducted in Bangladesh between 2009 and 2010 identified a total of 21 TB-positive cattle by postmortem examination, and two monkeys died due to pneumonia, of which *M. orygis* has been isolated from 18 cattle and two rhesus monkeys. Interestingly, 15 cattle and two monkey isolates had similar profiles based on multiple-locus variable-number tandem repeat analysis (MLVA). On the other hand, the remaining three cattle isolates had different MLVA patterns, and this shows the existence of strain diversity among *M. orygis* and its establishment over a period of time in the study area (26). *M. orygis* was isolated from a deer and a blue bull from a zoo in Nepal. Intriguingly, spoligotyping shows that spacer 3 was absent, although it is commonly reported in this species. This may be due to two point mutations in this spacer (15). *M. orygis* from bull and deer had similar patterns based on mycobacterial interspersed repetitive unit-variable number of tandem repeats (MIRU-VNTR) analysis of 22 loci. Thus, the same strain is circulating in captivity and acts as a source of infection to other susceptible animals through aerosol, contaminated food, and water (15).

In 2015, *M. orygis* was isolated and characterized from a free-ranging rhinoceros in Nepal. Dendrogram construction based on MIRU-VNTR results revealed that the rhinoceros isolate falls into a unique position and had a single difference in the MIRU 424 locus (13). Another study screened 3,581 cattle and buffaloes in slaughterhouses in Pakistan for bovine TB and 34 animals had TB-like lesions, of which 10 animals were found to be positive for *M. orygis* (34). In New Zealand, *M. orygis* infection was documented in cows and possibly transmitted from an animal attendant (29). Moreover, recently in India, *M. orygis* was isolated from cattle TB, which was found to be positive by comparative intra-dermal test (17). In the western hemisphere, *M. orygis* was recovered from a one-horned rhinoceros suffering from neural granulomatosis infection (38). Recently, in India, isolation of *M. orygis* was reported from two blackbucks, eight spotted deer, eight cattle, nine buffaloes, and one bison, and the species was confirmed by PCR amplification and whole-genome sequencing (37, 50). In another study, 567 samples were collected from 500 animals, including bulls, bull calves, and bullocks. Of these, 16 animals tested positive for MTBC cultures, with 13 of them identified as *M. orygis* (51). Considering all these reports, it is speculated that *M. orygis* infections in wild and domestic mammals have been underreported and received less attention. This may be due to a lack of an in-depth epidemiological surveillance mechanism and control program for both domestic and wild animals at the national level in many countries. At the field level, most of the animal TB cases are not identified up to the species level due to a lack of standard diagnostic assays to distinguish each member of MTBC.

## *M. orygis* in humans

Zoonotic tuberculosis is an extremely overlooked concept, and approximately 1.4% of cases of human TB were reported as zoonotic (52). The exact incidence rates of zTB remain uncertain because of the dearth of surveillance data in most countries. However, the data that are now available show that zTB has an impact on particular populations and environments (53). Previously, it was estimated that zTB was mainly caused by *M. bovis*, rarely by *M. caprae*, but in recent times *M. orygis* has also been included in the list (19, 54). In India, a total of 940 mycobacterial cultures from human TB patients were analyzed to estimate the zoonotic burden of animal-adopted MTBC, of which 0.7% (*n* = 7) isolates were *M. orygis* (Table 3). Interestingly, *M. bovis* was not identified in any of these cases (45). Between 2005 and 2010, a total of 1,763 patients from Australia were diagnosed with MTBC infection; of these, eight patients were infected with *M. orygis*, whereas *M. bovis* was identified in two cases (55). In New York, from 2005 to 2016, eight cases of *M. orygis* have been reported based on the whole-genome sequence analysis of 6,322 MTBC species (30). *M. orygis* was isolated from an immunocompetent patient suffering from lymphadenitis in New York (30) (Table 3). One case was reported in New Zealand, wherein a dairy handler was found to be infected with *M. orygis* (29). In the United Kingdom, out of 3,128 clinical MTBC isolates analyzed by SNP-IT tools, 24 were identified as *M. orygis* (14). Of these, two pairs of *M. orygis* were identical and thus speculate on the possibility of human-to-human transmission of this pathogen or common exposure to the same infected animal (14). Furthermore, *M. orygis* was isolated from five patients in Norway, and the author concluded that all five patients were from South Asian countries and imported *M. orygis* to Norway (56). In India, a study was conducted, and out of 1,105 patients, eight were infected with TB caused by *M. orygis*. Possible risk factors such as close contact with infected animals or consumption of unpasteurized animal products for acquiring zTB were not ruled out (33). We can speculate that the same species of *M. orygis* isolated from humans that was earlier identified in animals has raised concerns about the wide host spectrum of this pathogen. Recently, a study conducted in Canada found that out of 42 patients infected with animal lineages, 21 were infected with *M. orygis* (25). All these cases prompt the international health authorities to reconsider *M. bovis* as the sole cause of zTB. In the human health sector, the major focus is to detect and treat each TB case to achieve the goal of “ending TB, “rather than identifying each member of the MTBC. Data on zTB are only available from 61 countries (57), suggesting that the true extent of zTB in many developing and underdeveloped countries with large livestock populations and high TB burdens is likely underreported.

## Challenges and strategies to combat zoonotic tuberculosis

Despite being an ancient and curable disease, TB remains one of the world’s leading causes of mortality in humans. A target set by the WHO in 2018 is to reduce TB death and incidence rates by 95% and 90%, respectively, by 2035. Nevertheless, it is not possible to reach this milestone without adequate surveillance, monitoring, and control programs for zTB, including domestic and wild animals. In this study, we are highlighting some of the major challenges and areas to focus on for the control of zTB caused by *M. orygis* and *M. bovis*.

### Inability to differentiate MTBC

Routine laboratory diagnostics employed for the detection of zTB are inefficient to differentiate human- and animal-adopted MTBC species (32). At present, in most resource-limited countries, identification of TB causative agents is largely made by the detection of acid-fast bacilli in sputum smears (less sensitive test), chest X-ray for humans, and isolation of the etiological agent. The estimation of bacterial growth rate and phenotypical and biochemical characteristics is also a commonly employed method for the identification of MTBC (58, 59), while the tuberculin skin test remains the standard method for TB diagnosis in animals. However, commonly used comparative intradermal tuberculin test and gamma interferon release assays have limited sensitivity and specificity (60), which may reduce their preference among stakeholders. Furthermore, the skin tuberculin test cannot distinguish between *M. tuberculosis* and *M. bovis* infection (61). Conversely, these traditional techniques are very slow, time-consuming, non-reproducible, and not able to identify all the members of MTBC up to the species level.

Alternatively, techniques such as spoligotyping, IS*6110-*RFLP, MIRU-VNTR, analysis of SNPs, multilocus sequence typing, RD region analysis, and MALDI-TOF can be used for species-level identification, although these techniques have certain limitations and cannot always distinguish between each member of the MTBC. However, SNP analysis is capable of differentiating MTBC members, providing more species-level differentiation, but it involves several working steps and days to obtain the results. For example, a total of 44 *M. africanum* isolates from humans were spoligotyped, and two isolates showed a typical *M. orygis* spoligotyping pattern; however, authors grouped them under *M. africanum* subtype I (62). Commercially available Hain life science GenoType MTBC assay based on the *gyrB* gene also failed to differentiate *M. africanum* from *M. orygis*, *M. pinnipedii*, *M. suricattae*, and *M. mungi* (63). On the other hand, several single-tube multiplex RT-PCR targeting the presence and absence of RD regions is a viable option (22).

Furthermore, these molecular tests most often yield ambiguous results (64). For instance, a case was reported in 2010 wherein a patient was infected with zTB (*M. bovis*) and was misidentified as *M. tuberculosis* infection using a PCR assay targeting the *IS6110* insertion sequence. (65). Similarly, the IS*6110* PCR technique also failed to identify *M. bovis* in a patient suffering from vertebral spondylodiscitis (64) due to a lack of sufficient copies of the *IS6110* gene in *M. bovis*. Sometimes, it becomes more difficult for clinicians and veterinarians to identify and diagnose MTBC when it is co-infected with more than one mycobacterial species. Analysis of clinical samples (*n* = 331) from extra-pulmonary human TB patients and 52 bovine TB cases revealed that 29 (8.7%) of human samples and 35.7% (*n* = 20) of cattle were infected with *M. bovis* and *M. tuberculosis*, respectively (66). Given the limitations of current diagnostic techniques, it is possible to attribute some bovine TB infections to *M. orygis* rather than *M. bovis.* Despite extensive studies on MTBC for a long time, we do not have an appropriate method which is uncomplicated, rapid, affordable, and able to differentiate members of the MTBC. Misidentification may lead to the wrong treatment regimen and case mismanagement and may lead to the development of multidrug resistance. However, for both treatment and epidemiological studies, precise species identification of MTBC members is crucial (22). Moreover, a reliable method to differentiate these species can offer significant benefits in terms of personalized patient care, public health management, and disease surveillance.

For animal surveillance of zTB, indirect enzyme-linked immunosorbent assay (ELISA) and caudal fold test are practical methods for screening a large number of samples as they are cost-effective and quick. However, due to the 99.9% nucleotide similarity among MTBC members (5), many immunogenic antigens are highly conserved, making it challenging to identify subspecies-specific antigens for developing a diagnostic test specifically for *M. orygis*. Despite these challenges, recent advances in diagnostic biomarkers, comparative proteomics, transcriptomics, DNA methylomes, peptidomics, mass spectrometry, and *in silico* analysis offer promising opportunities to identify species-specific antigens that could aid in the detection and differentiation of MTBC (67). For example, peptidomic analysis of mycobacterial secreted proteins has the potential to identify the species (68), while computational analysis of *M. orygis* hypothetical proteins revealed two proteins, such as QOY47331.1 and QOY49361.1, which have diagnostic and therapeutic potential against *M. orygis* (69). However, further testing and validation with clinical samples are required. A similar approach has been employed to identify *M. bovis*-specific peptide (ESAT-6, CFP-10, and *Rv3615C*) antigens, which were tested in skin tests and showed promising results (70). Similar studies could lead to the identification of *M. orygis*-specific antigens that may be suitable for use in the caudal fold test.

For the precise diagnosis of *M. orygis* cases, a two-step protocol enabled provisional identification by PCR and assessment of inconclusive results through WGS, and SNP analysis can be employed with the help of unique genetic markers (24), depending on the needs and available resources. For this purpose, various SNPs unique to *M. orygis*, such as T to G at the 38th and 113th codon of *Rv2042C*, C to G at the 334th of *mmpS6*, and G to C at the 698th codon of *Rv0444c* mentioned in Table 2 and SNPs *PE35* (71), can be used for the development of *M. orygis*-specific RT-PCR. In conclusion, we recommend the identification of *M. orygis*-specific antigens to develop ELISAs and caudal fold tests for animal surveillance. For diagnosis, preliminary identification by PCR should be followed by further analysis of inconclusive results using WGS and SNP analysis. Furthermore, MTBC can be distinguished from non-tuberculosis mycobacteria (NTM) using a PCR assay based on the new short-chain dehydrogenases/reductases (SDR) gene (72) and surface plasmon resonance (SPR) biosensors based on organic light-emitting diode (OLED) (73).

### Fragile surveillance system

In the last 70 years, most of the emerging infectious diseases of humans arises from domestic or wild animal-adopted pathogens and accounted for more than 60% of human infections (74), of which zTB is an important devastating disease of humans and accounted for 1.4% (142,000 human cases) of the global TB burden (75). The percentage of zTB is underreported due to lack of national-level surveillance, specifically in developing and resource-limited countries. Moreover, surveillance initiated at the time of the disease outbreak also stopped once the situation came under control. For instance, in Africa, a weak surveillance system in animals led to 10–15% zTB due to *M. bovis* (76). In Italy, 27% of patients were underreported in a 10-year period (77). Between 2009 and 2014, in Southern Denmark, 7.5% of TB cases were underreported, and approximately 71.1% of TB cases were wrongly diagnosed (78). In the Pernambuco state of Brazil, 29% of TB cases were underreported, and most of them were co-infected with HIV, and according to the WHO, in Colombia, 30% of the cases were underreported (79, 80). Reports indicate that a study conducted in countries such as Cape Verde, Saudi Arabia, and Iran revealed that 40, 54.9, and 86.86% of human TB cases are underreported or not officially documented (81–83). Furthermore, most zTB surveillance systems are implemented independently for the animal and human health sectors, without an integrated strategy. Out of 119 WHO signatory nations, the majority (89.9%) of the countries lacked surveillance data about zTB, and in the majority of surveillance systems, the One Health Framework was not incorporated (84). Therefore, the true burden of zTB is underreported and understudied (53).

To understand the actual burden of animal TB, animal health surveillance in both slaughterhouses and the herd is required. Slaughterhouse surveillance will help in the improvement of inspection programs and has proven to be fruitful in many countries such as Canada, Denmark, and Northern Ireland (85–87). Surveillance of wildlife becomes even more challenging, but monitoring wild animals with modern technology can aid in surveillance efforts. Convolutional neural networks (CNNs) can be used to analyze images such as CT scans and X-rays to identify symptoms of TB in animals (88). Coyotes, Virginia opossums, wild and feral pigs, and other carrion-feeders have been employed as sentinels to offer broad surveillance coverage for TB in animal maintenance hosts (89–91). A study carried out in New Zealand employed captivity-raised feral pigs as sentinels for monitoring *M. bovis* in maintenance hosts, specifically brushtail possums (*Trichosurus vulpecula*). The study indicated that these pigs were an effective indicator of TB in wildlife (92). The TB surveillance programs should be maintained with enhanced trust to ensure the quality of animals and animal-based products and for human wellbeing. In addition, to increase the effectiveness of a surveillance system, frequent evaluation is also necessary. For instance, in Spain, even after the implementation of the test and slaughter policy, the disease still persists; therefore, a re-evaluation of the surveillance strategies needs to be in place (93). A well-planned surveillance system with clear objectives, case definitions, and diagnosis up to species level, active involvement of different stakeholders, and sharing of information with veterinary, human, and public health authorities are the cornerstones to combat TB in animals and humans. These measures require huge financial and human resource allocations, which are a challenging task for resource-limited countries. Indeed, this would help to understand disease trends and patterns and identify vulnerable target groups and reservoirs of TB.

### Wildlife reservoir host

The MTBC has a wide host range and hence can easily spill over into domestic animals, wild animals, and humans. There are several TB wildlife reservoirs, such as white-tailed deer, wild boar, badger, African buffalo, brushtail possum, and red deer (11). Reservoir hosts maintain the infection and spill over to other susceptible hosts, thus creating obstacles toward disease control and eradication (94).

Effective methods should be used to limit the transmission between domestic animals and humans from wild animals. Segregation by using barriers such as electric fencing and sheet metal gates has been proven to be helpful in disease transmission (95). According to the study in the UK, the badger is the major TB reservoir; therefore, using measures such as feed bins, electric fencing, and metal gates prevents the entry of badgers into cattle farm buildings, reducing the transmission between cattle and badgers (96). Similarly, one study in the US showed reduced transmission between cattle and white-tailed deer by fencing the buildings and using dogs for protection (97). Leaving and improper disposal of carcasses also leads to the risk of zoonotic disease transmission (98). More experimental research needs to be conducted in this area to analyze its effects and determine whether it is actually helpful in disease control or not.

### International trade of animals

International trading of wild and domestic animals is increasing due to the reduction in trade barriers, and it is one of the contributing factors that enhance the risk of zoonotic disease transmission (99). As trade demands close contact with handlers, buyers, etc., the likelihood of zoonotic infections increases. Annually, approximately 1.09 million cattle are imported from Mexico to the USA (94), and despite having proper TB eradication programs, nearly 91% (97/106) of human *M. bovis* isolates in the USA were of Mexican cattle origin (100). Similarly, an outbreak was reported in Trentino, Italy, due to the importation of cattle infected with *M. caprae* from Germany and Austria (101). Similarly, molecular characterization of bovine TB isolates from cattle in Morocco revealed that similar spoligotype patterns were also observed in Algeria, Spain, the US, and Argentina (102). This is probably due to the importation of cattle from Morocco to the US and Europe and the sharing of a border between Morocco and Algeria. The purchase of animals without screening is also responsible for this cross-border transmission between adjacent countries. Tuberculin skin testing (TST) should be done before import and export. However, sometimes, it gave false-negative results, as a study in Poland revealed that during the importation of *M. bovis-*infected alpaca from the UK, TST gave false-negative results (103). Fewer data are available for wildlife importation, but it is estimated that millions of wild animals were imported, mostly from South and East Asian countries, and responsible for disease transmission in animals (104). It would be difficult to predict the frequency of importation of TB-infected animals through animal trade. This may depend upon various factors such as high disease risk area, herd size, Btb prevalence status of import and export countries, and frequency of animal movement from non-officially tuberculosis free (OTF) countries to OTF countries (105).

To tackle this situation, translocation control is necessary, which prevents further disease emergence. A strategy like regionalization comprises zoning and compartmentalization (106) that ensures safe trade across and outside the country and minimizes the risk of disease transmission between animals and humans. Effective diagnosis methods before the import of animals and post-movement testing are also required. For importation, OTF states/countries may restrict the movement of animals from non-OTF states/countries and import animals only from low prevalence states/countries. Imported animals must have a certificate according to the guidelines of the World Organization for Animal Health (WOAH) indicating that they are free of TB, just like plants and their products require strict transportation laws, phyto-sanitary certification (107), and import inspections.

### Lack of interdisciplinary approach

Like other zoonotic diseases, zTB cannot be addressed by the human or veterinary health sectors acting alone; therefore, inter-sectoral collaborations from different disciplines at the national and international levels are required. Understanding the complex relationship between humans, animals, and the environment through integrated approaches such as One Health plays an important role in the surveillance, diagnosis, control, and prevention of zoonotic diseases. Though the One Health approach is an old concept due to lack of implementation, it does not get recognition. There have been several outbreaks of diseases that spread from infected birds and animals to humans, including West Nile virus, Nipah, Spanish flu, and COVID-19, that indicate a lack of One Health approach and adversely affect the human and animal health (108–110). In 2023, the G20 forum also highlights the importance of the One Health concept in TB control and management of anti-microbial resistance. *M. orygis* could be broadly distributed across South Asia and other parts of the world, highlighting the organism’s importance in the context of One Health (13).

There are some examples wherein the One Health approach helped to control diseases, such as, in Arizona, death in humans was reported due to Rocky Mountain spotted fever, which was transmitted from dogs to humans by tick bites. The collaboration between animal and public health officials reduced the disease risk by providing spray, a neuter clinic for dogs, and regular application of pesticides around homes. Similarly, in Egypt, Africa, and the Arabian Peninsula, the prediction of Rift Valley fever was done by using weather forecasting, which helped in the implementation of early mosquito control programs and thus reduced the health consequences (111). The veterinary and human health sectors worked together to control foodborne illnesses such as TB, brucellosis, typhoid fever, and diphtheria through the implementation of milk pasteurization from diseased cows, leading to the near eradication of these diseases in the USA. This is the best example of a One Health approach intervention in the 19th century (112). Recently, Martínez-Lirola et al. (113) reported the importance of a long-term, integrated One Health approach to identify the role of *M. caprae* in human TB cases that originated from animals in a region of Spain between 2003 and 2022. Hence, to address the challenges of zoonotic diseases, both public and animal health officials should work together and share information that helps raise awareness among the public, promote early diagnosis, and provide proper treatment. This will also reduce the duplicate efforts made by officials in disease control.

### Lack of funding, infrastructure, and policy

Despite many programs such as the Global Fund and national strategic plans, there is a lack of sufficient funding and policies, which prove to be an obstacle to disease elimination. Inadequate funding leads to insufficient infrastructure, lack of standard operating procedures (SOP), poor quality care, poorly trained technicians, poor authority functions, etc. (114). According to the WHO Report 2017, there was a lack of funding for health centers in developing nations such as India and Indonesia, which resulted in an underreporting of TB cases and treatment delays. India’s Revised National TB Control Programme (RNTCP) offers free treatment to TB patients. However, despite the increasing TB incidence rate, the program only receives 1% of the country’s GDP because of insufficient funding in the public health sector (115). On the other hand, in many veterinary practices, particularly for livestock and wildlife, there is limited access to laboratory facilities for animal TB detection, and there is no established policy in place to test Btb samples (116). Mostly, the funds flow from central to state government and then to the district level, because of which the officials delay in payment and several financial problems occur in TB control programs. Moreover, when it comes to bovine TB, most of the funding and donor agencies completely underrate the importance of zTB (117). There is a lack of an effective policy that explicitly mentions the guidelines regarding zTB.

Generally, in most of the developing countries, all the fund programs work on a performance-based funding concept that mainly depends upon the completion of targets, irrespective of the quality of care, cost-effectiveness, long-lasting impact, and effectiveness of the strategy (118). Thus, efforts should be made to find need-based permanent solutions. Hence, there is a need for adequate funding and a strong political willingness to provide all necessary resources. Global plans to eradicate TB mostly focus on developing vaccines, diagnostic tools, and drug development, assigning more funding for these areas than for operational research. Therefore, a new financing scheme for TB control is required for both biomedical and operational research. Furthermore, collaboration between the private and public sectors and integrating international or externally funded TB programs into national TB programs could offer effective resource allocation to national healthcare centers and accelerate progress.

### Lack of awareness

Providing treatment to patients is not the only aim of a TB eradication program, raising public awareness about the disease is also a crucial component of the program. People are re-infecting themselves because of knowledge scarcity about the disease and its treatment. Lack of awareness about zTB, disease transmission routes, major symptoms of TB, and accurate treatment for the disease is a major setback in most of the developing countries. For example, in a study in India, out of 200 patients, 170 were not aware of even the Directly Observed Treatment, Short Course Program (DOTS) (119). Most people are oblivious to the national programs organized by the local government. When it comes to zTB, farmers are unaware of the risk of zTB and where to dispose of the aborted, infected animals (120). One study in Nigeria showed that approximately 37.5% of livestock farmers were not aware of the risk factors associated with the consumption of unpasteurized milk and its products and modes of zoonotic transmission (121).

To raise public awareness regarding zTB, several educational campaigns should be conducted to disseminate information on current health policies, vaccines, personal hygiene, implications of pasteurization of milk and meat products, and the importance of treatment adherence and social distancing during infection. These educational campaigns use a variety of media, including books, billboards, radio, newspapers, posters, television, and social media (122). For instance, a study conducted in Delhi (India) has shown the impact of mass media awareness on TB among women, and they showed that billboards and television are potential tools for developing awareness strategies against TB (123). This can be exemplified by the fact that in India, the polio awareness and eradication program was initiated and promoted with considerable determination, which led to polio eradication in 2014; similarly, the zTB awareness program must be organized and implemented. Government officials should also implement TB awareness programs in educational institutions such as schools and colleges to enhance students’ understanding of the disease. This strategy can improve information retention, which students are likely to share with their family members.

## Conclusion and future perspectives

*Mycobacterium orygis* is a member of the MTBC, and its zoonotic potential is often underestimated. The One Health approach calls for enhanced vigilance and collaboration between medical, veterinary, and environmental disciplines to better understand the epidemiology, transmission, host range, reservoirs, and zoonotic potential of this pathogen. More research is needed to determine the global prevalence and burden of *M. orygis*. Developing rapid and accurate diagnostic tools and effective treatment protocols will be critical in mitigating the public health threat posed by *M. orygis*. There is a need for systematic surveillance of *M. orygis* in humans, animals, and the environment for early detection and control. Strengthening bio-safety measures in laboratories and educating health professionals and the public about the risks associated with zTB can help in managing this pathogen.
